# Immune cells composition in the skin and subcutaneous adipose tissue of patients with systemic sclerosis

**DOI:** 10.1111/ddg.15864

**Published:** 2025-11-20

**Authors:** Marija Geroldinger‐Simić, Ana E. Aguilar González, Gerald Exler, Georg Stary

**Affiliations:** ^1^ Department of Dermatology and Venereology Ordensklinikum Linz Elisabethinen Linz Austria; ^2^ Faculty of Medicine Johannes Kepler University Linz Austria; ^3^ Department of Dermatology Medical University of Vienna Wien Austria; ^4^ CeMM Research Center for Molecular Medicine Austrian Academy of Sciences Wien Austria; ^5^ Christian Doppler Laboratory for Chronic Inflammatory Skin Diseases Vienna Austria

**Keywords:** High‐content spectral flow cytometry, skin, subcutaneous adipose tissue, systemic sclerosis

## Abstract

**Background and objectives:**

Systemic sclerosis (SSc) is a rare, chronic autoimmune disease characterized by fibrosis of the skin and/or internal organs. Emerging evidence suggests that subcutaneous adipose tissue may contribute to systemic inflammation and fibrosis in SSc. This study aimed to conduct a comprehensive analysis of immune cell composition in SSc by simultaneously examining blood, skin, and subcutaneous adipose tissue.

**Patients and Methods:**

Using spectral flow cytometry, we profiled major immune cell subsets and explored their associations with clinical features of SSc.

**Results:**

Patients with mild skin fibrosis (low mRSS) exhibited increased cDC1, moDC, and ThGM‐CSF cells in the skin, alongside with an influx of Th22 cells and reduced terminal NK cells in subcutaneous adipose tissue. In SSc patients with lung fibrosis, peripheral blood showed decreased NK cells and increased CD8^+^ T cells. Anti‐Scl‐70‐positive patients demonstrated elevated CD8^+^ effector T cells, whereas anti‐centromere‐positive patients showed increased ThGM‐CSF cells in the skin.

**Conclusions:**

These findings highlight the potential role of distinct immune subsets for disease progression and tissue‐specific fibrosis in SSc.

## INTRODUCTION

Systemic sclerosis (SSc) is a systemic, chronic autoimmune disorder characterized by progressive fibrosis of the skin and/or internal organs, most notably the lungs and gastrointestinal tract. SSc is divided into two major subtypes based on the extent of skin involvement: limited cutaneous SSc (lcSSc) and diffuse cutaneous SSc (dcSSc).^1^ While both subtypes share common pathophysiological features, dcSSc is associated with a more progressive disease and higher risk of internal organ involvement, including severe lung fibrosis.^2^ The underlying pathogenesis of SSc is multifactorial, involving immune system dysregulation, vascular abnormalities, and excessive extracellular matrix deposition, all of which contribute to the fibrotic progression observed in the disease.^3^, ^4^

The immune system plays a central role in the pathogenesis of SSc, with both innate and adaptive immune components contributing to tissue injury and fibrosis.^3^ The early stages of disease are characterized by autoimmunity, with the production of autoantibodies such as anti‐topoisomerase I (anti‐Scl‐70), anti‐centromere antibodies (CENP), and/or anti‐RNA polymerase III antibodies, which are closely linked to specific clinical phenotypes and disease progression.^5^ Immune profiling studies in SSc have largely focused on systemic changes in the blood;^6^ however, tissue‐specific immune alterations in the skin and subcutaneous adipose tissue, which are direct sites of fibrosis, have received less attention. Recent studies suggest that the subcutaneous adipose tissue plays a crucial immunoregulatory role,^7^ and dysfunction of resident immune cells in the fat may contribute to systemic inflammation and fibrosis in SSc.^8^

The aim of this study is to perform a comprehensive analysis of immune cell composition in SSc patients by examining blood, skin, and subcutaneous adipose tissue. Using spectral flow cytometry to profile all major immune subsets, we investigated how immune cell alterations are associated with clinical parameters of SSc. We hypothesized that dysregulation of immune cell signature in the skin and subcutaneous adipose tissue in SSc patients plays a key role in fibrosis of the skin, while more systemic immune changes are associated with progression of disease and lung fibrosis. By grouping immune profiles with clinical disease parameters, we aim to provide novel insights into the immune mechanisms driving SSc fibrosis.

## PATIENTS AND METHODS

### Patient cohort and recruitment

Patients with SSc and healthy controls were recruited at the Department of Dermatology, Ordensklinikum Elisabethinen Linz, Austria. All patients and healthy controls participated in this study voluntarily and have given fully informed written consent. This study was approved by the ethics committee of Johannes Kepler University Linz, Austria (protocol 1108/2021 and amendments). The diagnosis of SSc was made according to the 2013 classification criteria established by the *American College of Rheumatology* (ACR) and the *European League Against Rheumatism* (EULAR). The inclusion criteria for patients with SSc were age between 18 and 90 years and a confirmed diagnosis of SSc. Each patient was characterized according to clinical parameters, including medical history, clinical status (modified Rodnan Skin Score [mRSS]), and laboratory tests (including autoantibody profiles). Exclusion criteria for the control group were SSc, metabolic syndrome, type 2 diabetes mellitus, and acute infections.

Routine clinical diagnostics included testing for a panel of clinical antibodies, such as anti‐nuclear antibodies (ANA) and ANA subsets (anti‐centromere, anti‐Scl70, anti‐Rnp/Sm, anti‐Rnp70, anti‐SSA/Ro, anti‐SSB/La, anti‐Sm antibodies), using standardized ELISA and immunofluorescence assays. Lung fibrosis (LF) was evaluated using high‐resolution computed tomography (HRCT) scans and pulmonary function tests, while pulmonary arterial hypertension (PAH) was assessed through stress echocardiography and right heart catheterization.

### Skin and subcutaneous fat biopsies and sample storage

Tissue samples (deep skin biopsies including adipose tissue) were taken from patients with SSc under local anesthesia using a 6mm punch (a routine procedure in the dermatology outpatient clinic). Two skin samples, one from affected skin and one from unaffected skin, were collected. The inner side of the upper arm or the abdomen (favorable fat distribution, low wound tension) were preferred as biopsy sites. Tissue samples from the control group were obtained from tissue material collected during routine skin surgeries (full‐thickness skin grafts, scar corrections, or flap surgery) and most of them were matched for the biopsy site. For the purposes of the study, skin samples including subcutaneous white adipose tissue were obtained from this surplus material for the control group. Subcutaneous fat was separated from the dermis using a scalpel. The biopsies were weighed and subsequently frozen in fetal bovine serum (FBS; Gibco, Cat. No. 10500064) containing 10% dimethyl sulfoxide (DMSO; Sigma, Cat. No. 317275‐500ML), and stored at −80 °C until analysis.

### Immune cell isolation from PBMCs

Whole blood was collected using BD Vacutainer Glass Cell Preparation Tubes (BD; Cat. No. 362782), and PBMCs were isolated by gradient centrifugation according to the manufacturer's protocol. The isolated PBMCs were frozen in fetal bovine serum (FBS) containing 10% dimethyl sulfoxide (DMSO) and stored at −80 °C until analysis.

### Sample preparation for flow cytometry

Skin biopsies were thawed at 37 °C, washed twice with phosphate‐buffered saline (PBS; Gibco, Cat: 14190144), weighed, and finely minced with a scalpel. The tissue was then digested using the Whole Skin Dissociation Kit, human (Miltenyi Biotec, Cat: 130‐101‐540), omitting enzyme P to preserve surface epitopes for flow cytometry. Subcutaneous fat biopsies were thawed at 37 °C and washed twice with PBS before weighing and further processing. The tissue was digested in a 2 mL tube containing collagenase IV (10 U/mL; Fisher Scientific, Cat: LS004189) and deoxyribonuclease I (10 mg/mL; Sigma‐Aldrich, Cat: DN25‐1G) in RPMI 1640 medium (Gibco, Cat: 52400‐025) supplemented with 5% FBS. Both skin and fat biopsies were digested overnight at 37 °C with gentle shaking.

After digestion, the cell suspensions were filtered through a 100 µm filter. The skin suspension was washed three times with 10 mL ice‐cold PBS supplemented with 10% FBS, while the fat suspension was washed three times with 10 mL ice‐cold PBS supplemented with 2% FBS. Skin cells were then centrifuged for 5 min at 300 g and 4 °C, and the pellet was resuspended in PBS for antibody staining. The fat cell suspension was centrifuged for 10 min at 800 g and 4 °C to remove floating adipocytes. The supernatant was discarded, and the remaining cells were resuspended in 30 mL ice‐cold PBS supplemented with 2% FBS and centrifuged again for 10 min at 800 g an 4 °C to isolate a pure stromal vascular cell fraction. The resulting pellet was resuspended in PBS for antibody staining.

PBMCs were thawed at 37 °C in 5 mL RPMI 1640, centrifuged for 5 min at 300 g, washed once with PBS, and resuspended in cold PBS for antibody staining.

### Flow cytometry

Cells were stained for 30 min at 4 °C in the dark in PBS containing all antibodies and a viability dye to assess cell viability (see supplementary Table S1 for antibodies and dilutions). Cells were then washed with PBS containing 2 mM EDTA (Thermo Fisher, Cat: 15575020) and 1% bovine serum albumin (BSA; Sigma, Cat: A2153), and resuspended in PBS containing 3 mM EDTA. Samples were directly acquired on a Cytek Aurora spectral analyzer equipped with five lasers (355 nm, 405 nm, 488 nm, 561 nm, and 640 nm). Surface staining was performed according to standard protocols using a panel of fluorochrome‐conjugated antibodies, each titrated to the optimal dilution for the respective tissue. To ensure accurate spectral unmixing, single‐stained reference controls for each fluorochrome, as well as autofluorescence controls for unstained cells, were acquired.

The flow cytometry panel was optimized for tissue and blood separately as cell numbers allowed and FMO (fluorescence minus one) stainings were performed for each tissue to determine staining specificity.

### Statistical Analysis

Flow cytometry data was analyzed in FlowJo (version 10.8.1) and GraphPad Prism (version 10.0.3). Statistical significance was assessed using Two‐Way ANOVA with Tukey correction for multiple hypothesis testing. Normality testing was performed using Shapiro‐Wilk test in GraphPad Prism. Two‐way ANOVA was chosen as patient groups and immune cell subsets were compared within the same table and the Two‐way ANOVA allows for multiple comparison across rows.

## RESULTS

### Patient cohort

The study group included 13 patients with SSc and 13 controls without SSc. The median age of SSc patients was 59 (range 48–79) and nine out of 13 SSc patients were female. Six SSc patients had limited cutaneous SSc (lcSSc) and seven SSc patients had diffuse cutaneous SSc (dcSSc). The median disease duration in SSc patients was 6 years (range 2–38). ANA antibodies were positive in twelve out of 13 SSc patients, five SSc patients were positive for anti‐Scl70‐antibodies and four SSc patients showed positivity towards CENP. The characteristics of the patients with SSc and control group (without SSc) are summarized in Table 1.

**TABLE 1:** Overview of patient cohort.

Study group	SSc patients	lcSSc	dcSSc	Controls
Number, N	13	6	7	13
Female/ male, N	9/ 4	5/ 1	4/ 3	6/ 7
Age, median (range)	59 (48‐79)	64 (55‐79)	56 (48‐67)	70 (62‐88)
Limited SSc/ diffuse SSc, N	6/ 7			
Disease duration, Y median (range)	6 (3‐38)	7 (3‐24)	6 (3‐16)	
ANA positive, N	12	6	6	
Anti‐Scl70/ CENP, N	5/ 4	2/ 2	3/ 2	
mRSScore (0‐51), median (range)	8 (4‐44)	5 (4‐11)	16 (7‐44)	
Clinical manifestations				
Calcinosis cutis (CC), N	6	3	3	
Digital ulcers (DU), N	3	0	3	
Dysphagia, N	12	6	6	
Lung fibrosis (LF), N	6	2	4	
PAH, N	3	1	2	
Raynaud symptoms, N	11	5	6	
Reflux, N	7	4	3	
Sicca, N	7	3	4	
Mycophenolate mofetil, N	4	1	3	
Bosentan, N	4	1	3	
Macitentan, N	3	0	3	
Methotrexate, N	6	2	4	
Nintedanib, N	1	0	1	
Prednisolone, N	3	1	2	
Sildenafil, N	3	0	3	

### Immune compositional changes in SSc patients

Biopsies of lesional and non‐lesional skin as well as the adjacent subcutaneous adipose tissue along with a blood sample from SSc patients and healthy controls were taken for downstream analysis of immune cell composition. PBMCs were isolated from blood samples and tissue samples were digested. Subsequently, all samples were analyzed by spectral flow cytometry using a 32‐color panel spanning all major immune cell subsets in the blood, skin and fat (online supplementary Figure S1a–d, online supplementary Table S1, online supplementary Table S2).

**FIGURE 1:**
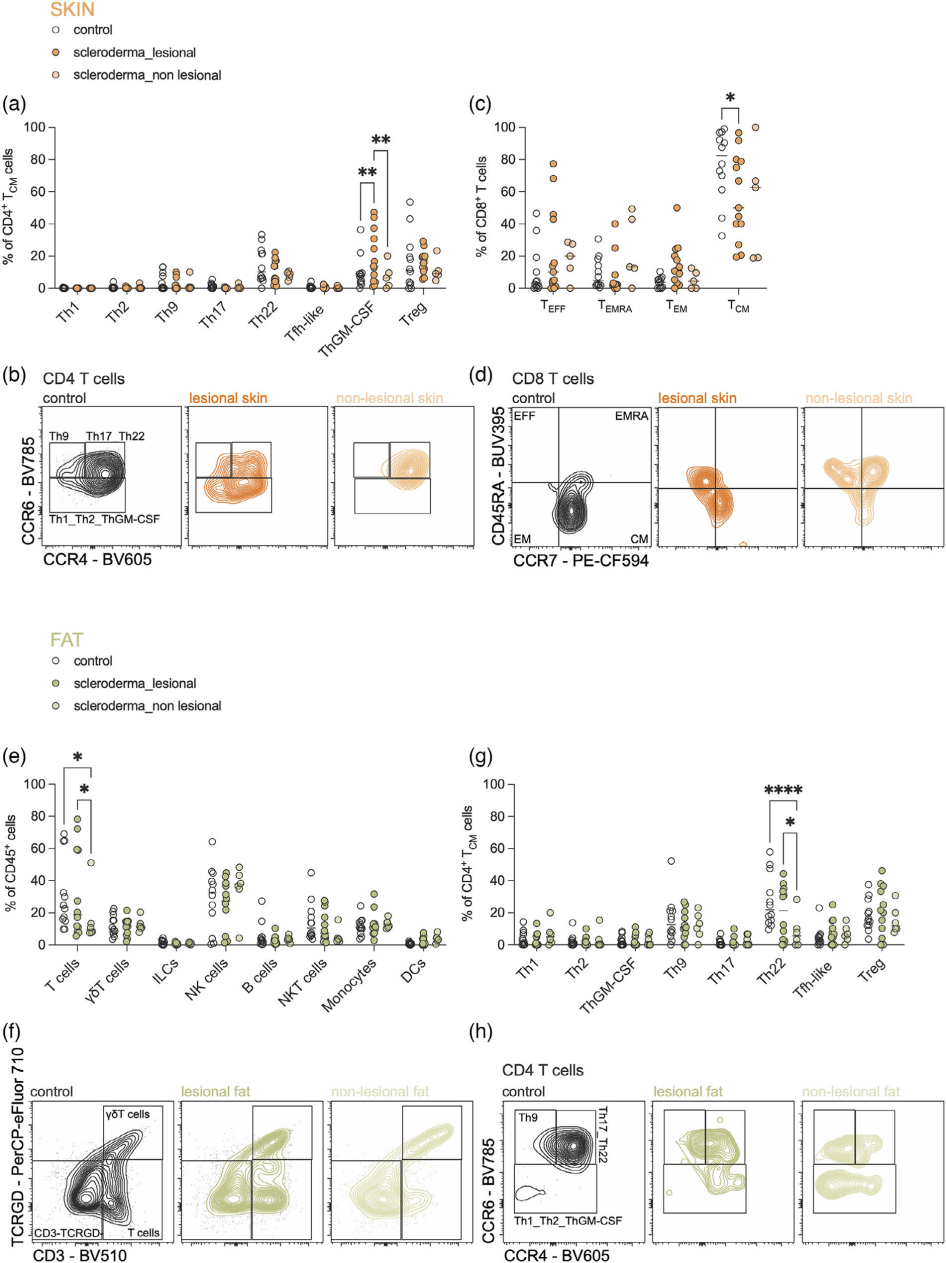
Changes in immune cell composition in the skin and subcutaneous fat of patients with systemic sclerosis (SSc).

To better understand systemic and tissue‐specific immune changes in SSc, we began by examining the blood for differences in immune cell composition between SSc patients and healthy controls. We observed an increase in CD4^+^ T cells in SSc patients, while NK cells were overall depleted (online supplementary Figure S2a). Notably, within the NK cell population, mature NK cells were relatively enriched in SSc patients compared to controls (online supplementary Figure S2b). While blood analysis provided insights into systemic immune activation, we extended our investigation to the skin and the adjacent subcutaneous adipose tissue, which has known immunoregulatory functions and resident immune cells in fat can be dysfunctional during inflammation.^9^ In lesional skin of SSc patients, we found a significant increase in pro‐inflammatory helper T cells of the ThGM‐CSF subset (Figure 1a,b) and a reduction in CD8^+^ central memory (CM) T cells (Figure 1c,d). Interestingly, differences in immune cell composition in subcutaneous fat were evident primarily in non‐lesional areas compared to healthy controls. These areas showed an overall decrease in T cell populations within adipose tissue (Figure 1e,f), with a marked reduction of Th22 cells (Figure 1g,h). Since Th22 cells are known to play a critical role in wound healing in the skin,^10^ their loss in non‐lesional adipose tissue may predispose these tissue sites to inflammation, fibrosis and ulceration, potentially exacerbating disease pathology.

### Mild skin fibrosis is associated with an influx of pro‐inflammatory cells in the skin

**FIGURE 2:**
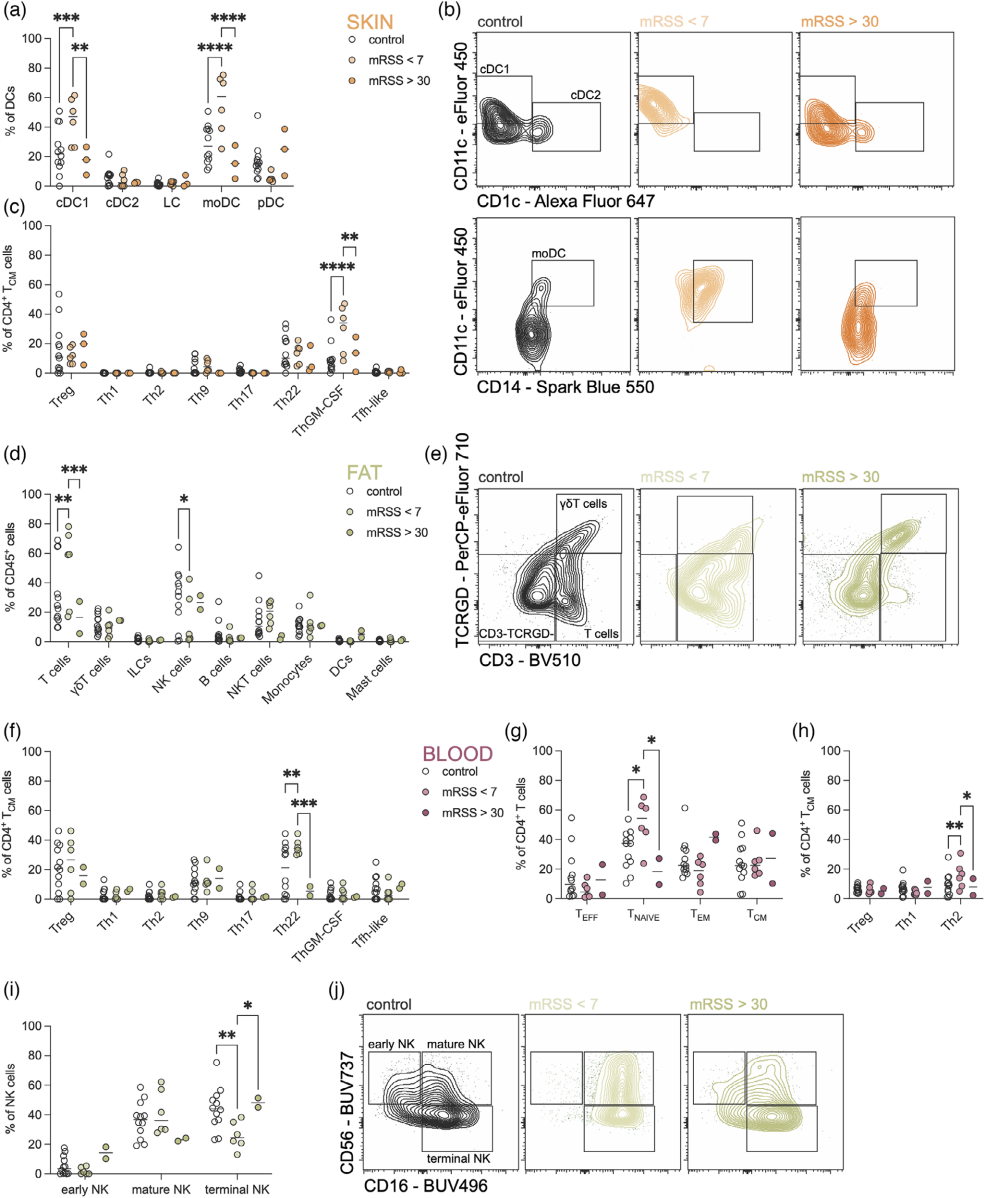
Mild skin fibrosis is associated with an influx of proinflammatory cells in the skin.

The minimal changes observed in the immune compartment when comparing SSc patients to healthy controls likely reflect the heterogeneity of the disease and the patient cohort. To identify more meaningful immune alterations, we grouped patients based on clinical parameters, focusing on the modified Rodnan skin score (mRSS), a standard measure of skin thickening that correlates with fibrosis and disease activity in SSc.^11^ In our cohort, patients with low mRSS (< 7) had a significant increase in conventional type 1 dendritic cells (cDC1) and monocyte‐derived DC (moDC) in the skin as compared to controls (Figure 2a,b). cDC1 and moDC are considered as mediators of systemic inflammation by driving Th1 and Th17 responses.^12^, ^13^, ^14^, ^15^, ^16^ Accordingly, we also found an elevation of ThGM‐CSF cells (Figure 2c) in the skin, which support differentiation and activation of myeloid cells among other pro‐inflammatory roles.^17^, ^18^ In SSc patients with mild skin fibrosis (reflected by a low mRSS), these cells could recruit adaptive pro‐inflammatory cells, thereby driving disease. This is reflected in subcutaneous fat, where an influx of T cells and Th22 cells (Figure 2d–f) was noted. In the blood, we observed an increase of naïve CD4^+^ T cells (Figure 2g), and Th2 cells (Figure 2h). Further, we found a decrease in terminal NK cells in the subcutaneous fat of patients with low mRSS (Figure 2i,j). Terminal NK cells have been shown to have anti‐fibrotic roles,^19^ therefore the loss of these cells could contribute to initiation of a systemic pro‐inflammatory response as seen in SSc.

Together, these results suggest that mild skin fibrosis, reflected by a low mRSS, is characterized by increased recruitment and activation of inflammatory cells mediated by cDC1 and moDC in the skin. This process might draw additional immune cells from adipose tissue and the bloodstream, setting the stage for fibrosis progression.

### Lung involvement of SSc is mainly reflected by immune cell changes in the peripheral blood

**FIGURE 3:**
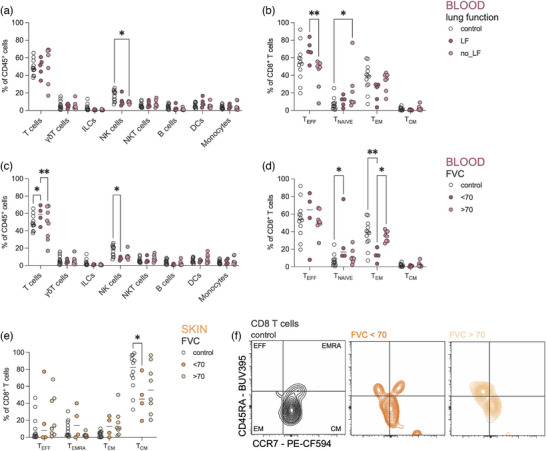
Lung involvement in systemic sclerosis is mainly reflected by immune cell changes in the peripheral blood.

While in patients with mild skin fibrosis (low mRSS) immune alterations were primarily observed in the skin and subcutaneous adipose tissue, patients suffering from lung fibrosis (LF), displayed the majority of changes in the blood. Patients with LF had a systemic decrease in NK cells in peripheral blood (Figure 3a), but an increase in CD8^+^ effector T cells compared to patients without LF (Figure 3b), highlighting how the later stages of disease are driven by changes in the adaptive arm of the immune system. Along the same line, patients with decreased lung function (forced vital capacity [FVC] < 70) also showed an increase of T cells in the blood (Figure 3c). In this group, specifically CD8^+^ naïve T cells are increased at the expense of CD8^+^ effector memory (EM) cells, which are depleted in the blood of patients with a FVC < 70 (Figure 3d). In the skin, patients with reduced lung function show a reduction of CD8^+^ CM T cells (Figure 3e,f).

These findings highlight the role of NK cells and CD8^+^ T cells observed in the blood during advanced disease, suggesting that adaptive immunity becomes increasingly pivotal in mediating fibrosis as SSc progresses.

### SSc‐specific autoantibodies are differentially associated with immune cell composition in the skin

**FIGURE 4:**
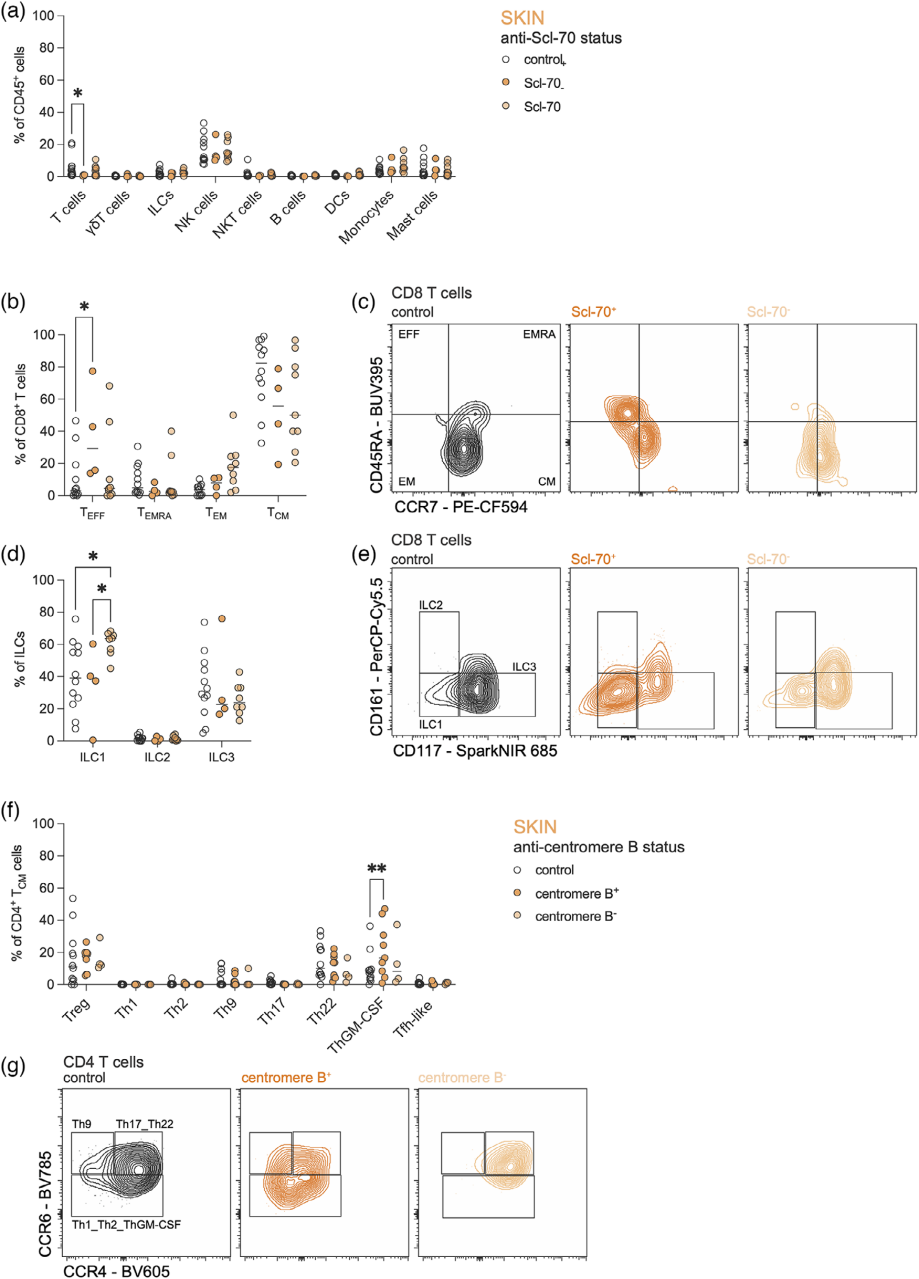
SSc‐specific autoantibodies are differentially associated with immune cell composition in the skin.

One of the key immunological features in SSc is the presence of specific autoantibodies, such as anti‐Scl‐70, CENP, and/or anti‐RNA polymerase III.^5^ Patients positive for anti‐Scl‐70‐antibodies showed a global loss of T cells (Figure 4a), but an increase of CD8^+^ effector T cells in the skin as compared to controls (Figure 4b,c). Compared to patients negative for anti‐Scl‐70, these patients also displayed a decrease of ILC1 in the skin (Figure 4d,e). On the other hand, patients positive for CENP, showed a significant increase in ThGM‐CSF cells in the skin (Figure 4f,g), which aligns with the notion that these patients often suffer from lcSSc,^5^ indicating that ThGM‐CSF cells are associated with milder course of SSc disease. The distinct immune profiles in the skin of SSc patients based on autoantibody status underline the heterogeneity of the disease.

## DISCUSSION

This study provides an in‐depth analysis of the immune cell composition in blood, lesional skin, non‐lesional skin, and subcutaneous adipose tissue in patients with SSc. While we acknowledge the small sample size used here, we still found significant differences matching with clinical parameters. We highlight significant changes in immune cell subsets across these different clinical parameters which are associated with stages of disease progression. The findings underscore the complexity of immune cell dysregulation in SSc and its potential implications for understanding disease progression, tissue fibrosis, and the role of specific immune subsets in modulating the disease.

One of the major observations in this study is the shift in the immune cell composition in patients with mild skin fibrosis (low mRSS), which is characterized by systemic immune activation and localized changes in the skin and subcutaneous fat. The increase in cDC1 and moDC in the skin of these patients is notable as both cell types are known to play critical roles in activating Th1 and Th17 cells,^12^, ^13^, ^14^, ^15^, ^16^ which are key drivers of inflammatory responses in autoimmune diseases. A previous study showed that moDCs induced T cell polarization towards Th2 and Th17, especially in the early stages of dcSSc.^20^ The early infiltration of pro‐inflammatory ThGM‐CSF cells in the skin further supports the idea that these cells contribute to the activation of myeloid cells and the recruitment of inflammatory cells from the periphery, exacerbating local tissue inflammation. Moreover, it has been shown that GM‐CSF stimulates fibrosis in SSc.^21^, ^22^ Accordingly, we also found a loss of Th22 in non‐lesional subcutaneous adipose tissue supporting an initiation of an inflammatory response in local tissues. Th22 cells are known for their anti‐inflammatory properties,^10^ and their depletion may promote a more pro‐inflammatory state in the fat tissue, potentially priming the skin and fat for further fibrotic progression. This aligns with previous studies suggesting that Th22 cells play a role in modulating adipose tissue inflammation and metabolic dysfunction.^23^, ^24^, ^25^ Contrarily we found an increase of Th22 cells in subcutaneous adipose tissue in patients with low mRSS. Interleukin (IL)‐22 has been shown to amplify fibroblast responses to TNF, potentially fostering skin fibrosis.^26^, ^27^ However, as our results on T helper cell subsets are based on surface marker expression, they need to be taken with caution and validated in future studies with evaluation of cytokine production. Consistent with these results, we also found a decrease in terminal NK cells in the subcutaneous fat of SSc patients with low mRSS. Terminal NK cells have been shown to have anti‐fibrotic roles,^19^ therefore their loss in early‐stage of SSc could facilitate the onset of inflammation and fibrosis in both the skin and adipose tissue, contributing to disease pathogenesis.

In patients with LF or reduced FVC, the immune alterations were predominantly seen in the blood, underscoring the systemic nature of disease progression. A reduction in NK cells in the peripheral blood of patients with LF aligns with previous findings of NK dysfunction in patients with autoimmune diseases,^28^ and also in patients with SSc.^29^ Moreover, studies showed that NK cells in SSc had altered cytokine production and reduced cytotoxic activity.^30^, ^31^ Beyond their cytotoxic role, NK cells also possess anti‐fibrotic properties, such as suppressing liver fibrosis.^32^ Their depletion could also lead to unchecked activation of immune cells like CD8^+^ T cells, which were increased in the blood of patients with lung involvement. This suggests that as SSc progresses, immune dysregulation shifts from localized tissue‐specific responses to systemic activation, with significant implications for organs like the lungs.

A key aspect of SSc pathogenesis is the presence of autoantibodies.^1^, ^5^ Distinct immune signatures were observed in patients positive for anti‐Scl‐70 or CENP. Patients with anti‐Scl‐70 positivity displayed an increase in CD8^+^ effector T cells in the skin which could indicate that autoantibody‐mediated immune activation is contributing to the progressive tissue damage seen in dcSSc, where fibrosis and systemic involvement are more severe.^33^ Previous studies showed that CD8^+^ T cells in SSc patients produce high levels of profibrotic IL‐13, enhance collagen synthesis and play a key role in early‐stage skin fibrosis and inflammation.^34^, ^35^ Conversely, CENP‐positive patients, who typically present with lcSSc,^33^ showed an increase in ThGM‐CSF cells in the skin, further suggesting that these cells play a key role in the milder course of SSc. This difference in immune profiles between anti‐Scl‐70 and CENP‐positive patients reinforces the idea that autoantibodies can direct the immune response in SSc, shaping the clinical presentation and influencing the immune cell landscape in different tissue compartments. Thus, this study highlights the importance of considering autoantibody status in conjunction with immune cell profiling to better understand disease pathogenesis and progression in SSc.

While further studies are needed to validate these findings in a larger patient cohort, this study provides a detailed snapshot of the immune landscape in SSc, revealing significant alterations in immune cell subsets across different tissues, including the skin, subcutaneous adipose tissue, and blood. These changes are linked to key clinical parameters such as skin fibrosis, lung involvement, and autoantibody status, highlighting the complex interplay between the innate and adaptive immune systems in driving disease progression.

There are several limitations of this study. A major limitation is the small sample size of the SSc cohort that warrants validation of our results in a larger patient cohort. Also, insufficient matching of sex and age between patients and controls, and the absence of analyses for monocyte subsets as well as the limited knowledge of the influence of medication on changes in immune cell profiles limit the conclusions of our study. Finally, we can only present relative values of cell subsets due to the study design and not using counting beads.

## FUNDING

Research Funding of the Medical Society of Upper Austria (MGS), Austrian Science Fund (Grant‐DOI: 10.55776/PAT8019123) and LEO Foundation (LF‐OC‐24‐001518) (both GS).

## CONFLICT OF INTEREST STATEMENT

None.

## Supporting information



Supplementary information
