# Supplementary material for: Immune cells composition in the skin and subcutaneous adipose tissue of patients with systemic sclerosis
Source: J Dtsch Dermatol Ges. 2025 Nov 20;24(4):482–92. doi: 10.1111/ddg.15864 (PMC13059067; doi:10.1111/ddg.15864)
Supplement: Supplementary file 1 — Supplementary information [file DDG-24-482-s001.docx]

[[Online-Supplement]]


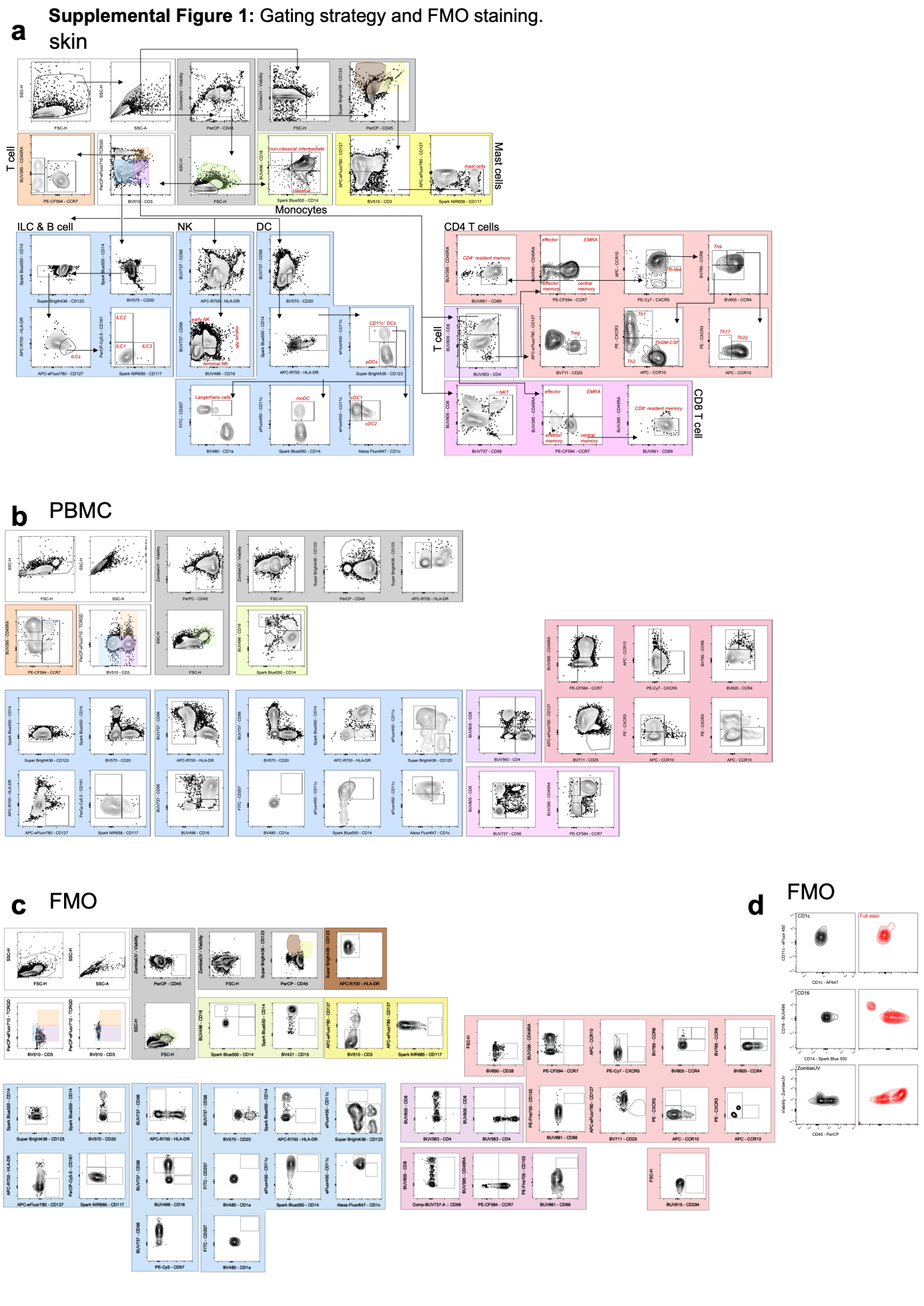


FIGURE S1 Gating strategy and FMO staining.


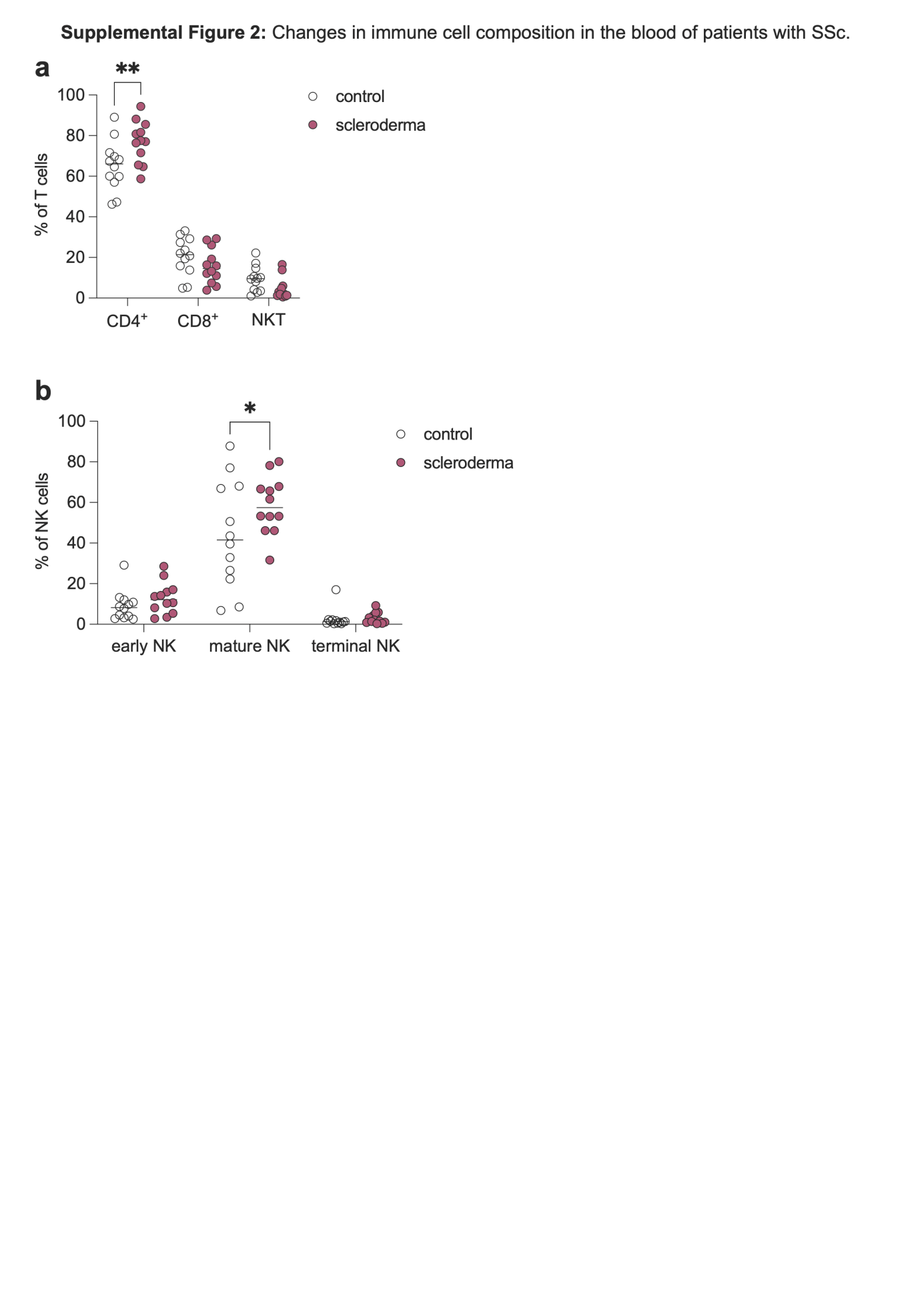


FIGURE S2 Changes in immune cell composition in the blood of patients with systemic sclerosis.

TABLE S1 Antibodies and dilutions used for sample analysis.

Table S1

| **Marker** | **Fluor** | **Clone** | **Supplier** | **Cat#** | **Dilution 1:X**  **(blood/tissue)** | **Laser** |
| --- | --- | --- | --- | --- | --- | --- |
| CD45RA | BUV395 | 5H9 | BD | 740315 | 400 / 400 | UV2 |
| Viability | ZombieUV | N/A | Biolegend | 423107 | 1000 / 1000 | UV6 |
| CD16 | BUC496 | 3G8 | BD | 612944 | 400 / 400 | UV7 |
| CD4 | BUV563 | OKT4 | BD | 750979 | 400 / 200 | UV9 |
| CD294 | BUV615 | BM16 | BD | 751216 | 200 / 400 | UV10 |
| CD69 | BUV661 | FN50 | BD | 750213 | 100 / 400 | UV11 |
| CD56 | BUV737 | NCAM 16.2 | BD | 564447 | 200 / 50 | UV14 |
| CD8 | BUV805 | SK1 | BD | 612889 | 200 / 400 | UV16 |
| CD15 | BV421 | W6D3 | BD | 740086 | 200 / 200 | V1 |
| CD123 | SuperBright 436 | 6H6 | ThermoFisher | 62-1239-42 | 100 / 200 | V2 |
| CD11c | eFluor 450 | 3.9 | ThermoFisher | 48-0116-42 | 200 / 200 | V3 |
| CD1a | BV480 | HI194 | BD | 566147 | 100 / 100 | V5 |
| CD3 | BV510 | SK7 | Biolegend | 344828 | 400 / 200 | V7 |
| CD20 | BV570 | 2H7 | Biolegend | 302332 | 200 / 100 | V8 |
| CCR4 | BV605 | L291H4 | Biolegend | 359418 | 50 / 100 | V10 |
| CD28 | BV650 | CD28.2 | Biolegend | 302946 | 100 / 100 | V11 |
| CD25 | BV711 | 2A3 | BD | 563159 | 100 / 100 | V13 |
| CCR6 | BV785 | G034 | Biolegend | 353422 | 50 / 20 | V15 |
| CD207 | FITC | MB22-9F5 | Miltenyi Biotec | 130-098-349 | 100 / 100 | B2 |
| CD14 | SparkBlue 550 | 63D3 | Biolegend | 367148 | 200 / 100 | B3 |
| CD45 | PerCP | HI30 | Biolegend | 304026 | 400 / 200 | B8 |
| CD161 | PerCP-Cy5.5 | HP-3G10 | Biolegend | 339908 | 200 / 400 | B9 |
| TCRGD | PerCP-eFluor 710 | B1.1 | ThermoFisher | 46-9959-42 | 200 / 400 | B10 |
| CXCR3 | PE | 1C6 | BD | 557185 | 50 / 50 | YG1 |
| CCR7 | PE-CF594 | 150503 | BD | 562381 | 100 / 100 | YG3 |
| CD57 | PE-Cy5 | NK-1 | SouthernBiotech | 9665-13 | 100 / 50 | YG5 |
| CD103 | PE/Fire700 | Ber-ACT8 | Biolegend | 350240 | 100 / 200 | YG7 |
| CXCR5 | PE-Cy7 | J252D4 | Biolegend | 356924 | 200 / 200 | YG9 |
| CCR10 | APC | 314305 | R&D | FAB3478A-100 | 50 / 100 | R1 |
| CD1c | Alexa Fluor 647 | L161 | Biolegend | 331510 | 100 / 200 | R2 |
| CD117 | SparkNIR 685 | 104D2 | Biolegend | 313250 | 200 / 200 | R4 |
| HLA-DR | APC-R700 | G46-6 | BD | 565127 | 200 / 200 | R5 |
| CD127 | APC-eFluor 780 | RDR5 | ThermoFisher | 47-1278-42 | 200 / 50 | R7 |

TABLE S2 Absolute number of CD45⁺ cells/mg.

**Table S2:**

|  | **Skin** | **Fat** |
| --- | --- | --- |
| C1 | 1,117.6 | 238.9 |
| C2 | 1,638.6 | 113.1 |
| C3 | 371.3 | 134.6 |
| C4 | 3,939.2 | 116.0 |
| C5 | 49.7 | 42.0 |
| C6 | 815.1 | 235.0 |
| C7 | 318.6 | 149.6 |
| C8 | 1,433.7 | 299.2 |
| C9 | 1,392.3 | 211.3 |
| C10 | 1,398.8 | 25.3 |
| C11 | 5,956.8 | 272.6 |
| C12 | 1,245.4 | 123.1 |
| SSd lesional 1 | 712.3 | 127.2 |
| SSd lesional 2 | 3030.0 | 18.9 |
| SSd lesional 3 | 463.3 | 38.0 |
| SSd lesional 4 | 2,111.9 | 160.7 |
| SSd lesional 5 | 2,349.6 | 185.5 |
| SSd lesional 6 | 2,791.2 | 260.2 |
| SSd lesional 7 | 1,500.5 | 436.5 |
| SSd lesional 8 | 1,149.2 | 22.3 |
| SSd lesional 9 | 1,248.9 | 104.0 |
| SSd lesional 10 | 5,843.0 | 80.7 |
| SSd lesional 11 | 1,330.9 | 1131.6 |
| SSd lesional 12 | 622.4 | 297.3 |
| SSd lesional 13 | 2,656.6 | 107.8 |
| SSd non-lesional 1 | 5,063.4 | 187.0 |
| SSd non-lesional 2 | 1,030.2 | 493.9 |
| SSd non-lesional 3 | 521.5 | 84.5 |
| SSd non-lesional 4 | 703.9 | 482.5 |
| SSd non-lesional 5 | 3,023.0 | 149.2 |

*Abbr.:* C, control; SSd, systemic sclerosis
